# Evolution of body image across treatment eras: a systematic review in young people and adults living with cystic fibrosis

**DOI:** 10.1183/16000617.0238-2025

**Published:** 2026-04-13

**Authors:** Cian Greaney, Natalia Cichy, Grace O'Sullivan, Darren Sills, Roisin Cahalan, Audrey Tierney

**Affiliations:** 1 School of Allied Health, University of Limerick, Limerick, Ireland; 2 Food, Diet and Nutrition Research Group, Health Research Institute, University of Limerick, Limerick, Ireland; 3 Nutrition and Dietetics, Nottingham University Hospitals NHS Trust, Nottingham, UK; 4 Physical Activity for Health Research Centre, Health Research Institute, University of Limerick, Limerick, Ireland; 5 Centre for Implementation Research, Health Research Institute, University of Limerick, Limerick, Ireland; 6 Discipline of Food, Nutrition and Dietetics, La Trobe University, Melbourne, Australia

## Abstract

**Objectives:**

Advancements in treatments for cystic fibrosis (CF) have improved prognosis, but variant-specific therapies have also driven weight gain. Traditional challenges to maintain weight are now less prevalent, with new body image concerns potentially emerging. This review aims to synthesise quantitative and qualitative evidence on body image in adults and young people (aged ≥16 years) with CF.

**Methods:**

Systematic search of MEDLINE, Embase, the Cumulative Index to Nursing and Allied Health, the Allied and Complementary Medicine Database and the Cochrane Library (January 2011 to August 2025) using CF-related keywords including body image and physical appearance.

**Results:**

Searches retrieved 29 eligible studies (26 quantitative, two qualitative, one mixed methods). The Cystic Fibrosis Questionnaire – Revised (CFQ-R) and Cystic Fibrosis Quality of Life Questionnaire (CFQoL) questionnaires assessed body image quantitatively (0–100). 15 studies reported CFQ-R body image scores (58±19 to 78±23) and five reported CFQoL body image scores (21±26 to 76). Pre-modulator era (≤2012) scores ranged from 64±27 to 70±27; initial rollout phase (2012–2019) scores ranged from 58±19 to 81 (post-ivacaftor); and in the widespread modulator era (2020 to present) were 63±32 to 67 (31). Gender effects were inconsistent. Higher body mass index, weight, and body fat were linked to better body image in some studies; psychological wellbeing and lower anxiety/depression consistently correlated with body image. Ivacaftor and life coaching improved body image, while elexacaftor/tezacaftor/ivacaftor, cognitive behavioural therapy, and self-management programmes showed no effect. Functional health was strongly linked to body image.

Qualitative findings highlighted influences of gender, weight change, functional and mental health, social support, early eating experiences, and limited clinician-initiated body image discussions.

**Conclusion:**

This is the first systematic review of body image in CF since new drug regimens emerged. Emerging body image disturbance patterns highlight the need for clinical strategies to screen for and address body image issues in CF care.

## Shareable abstract

**As treatments for cystic fibrosis (CF) improve, new challenges around body image are emerging. The review findings show body image is shaped not just by weight, but by mental health, function and support, highlighting the need for holistic CF care.** https://bit.ly/4jPk99w

## Introduction

Cystic fibrosis (CF) is an inherent, life-limiting disorder resulting from mutations in the CF transmembrane conductance regulator (CFTR) gene [1]. The disorder is characterised by consistent lung infections which result in poorer pulmonary function and a shorter life expectancy [2]. In recent years, improvements to clinical practices and drug therapies have altered the trajectory of CF prognosis [3]. Variant-specific therapies (VSTs) like CFTR modulator therapy have played a prominent role in altering the lives of people living with CF [4], with median survival now exceeding 50 years in many countries [5].

With this, novel complexities have surfaced, including increased rates of overweight and obesity (40.9%) [6]. This surge has coincided with the increased availability and use of VSTs, with evidence that VSTs reduce resting energy expenditure [7] as well as increasing the assimilation of food and nutrients [8, 9]. The most recently published European CF dietary guidelines and recommendations from the Academy of Nutrition and Dietetics outline that the conventionally advocated diet for people living with CF (*i.e.* high-energy, high-fat diet) no longer has sufficient clinical evidence to remain at the forefront of CF nutrition guidelines [2, 10]. This evolution coincides with evidence that diets of people living with CF are of poor quality and are dependent on energy-dense nutrient poor foods to achieve elevated energy targets [11]. The combination of inadequate diets, frequent use of VSTs [6], and the subsequent continued growth of overweight and obesity [6], suggests that these trends are likely to persist. This may mark a shift in CF care from navigating malnourishment to managing weight gain in many people living with CF.

Historically, the CF phenotype made achieving a healthy body mass index (BMI) a clinical priority [12–16], but more recent evidence has begun emphasising body composition alongside BMI, as measures of fat mass and fat-free mass are more strongly associated with respiratory outcomes, and low or normal BMI can conceal high fat mass or low fat-free mass [2]. Considering the concerns around weight gain and diet quality, CF is positioned as a condition that increases vulnerability to eating disorders and poor body image [17], and thus, the topic of body image in the new era of CF where overweight and obesity persists is of clinical relevance. Body image represents an individual's experience regarding their own embodiment, including appearance, function and perceptions of health [18], with negative body image in young adults associated with less healthy dietary habits, diminished physical activity, and worse psychological outcomes (*e.g.* anxiety and depression) [19], all of which are relevant in the CF context.

Sociocultural ideals emphasise muscularity for men and thinness for women [20–22]. In the context of CF, these gendered ideals interact uniquely with the traditional physical phenotype of the condition (*i.e.* frailer, underweight appearance). The first critical review on body image in CF [23] was published in 2012 and illustrated that female people living with CF often preferred lower weight, aligning with cultural ideals, despite health risks [24]. Conversely, male people living with CF were more likely to report dissatisfaction with their physique, driven by a desire to appear larger and more muscular. As a result, male people living with CF were generally more motivated to follow nutritional recommendations aimed at weight gain [23]. In the general population, body image disturbances are more common in females, although rates in males are rising [22]. In the modulator era of CF, it remains unclear whether these body image patterns will persist, or whether new body image disturbances will emerge related to increases in weight and BMI.

Given that the last body image review predated widespread modulator use and accelerated rises in obesity rates, an updated review on body image in CF is warranted. Furthermore, as rates of overweight and obesity begin to normalise and align with the general population [6], it may lead to an era in CF with evolving and increasing incidences of body image disturbances. The aim of this review is to comprehensively evaluate and collate findings on body image in CF. In doing so, this review could inform best practice approaches to address body image disturbances among people living with CF.

## Methods

### Search strategy and study selection

This systematic literature review was completed adhering to the Preferred Reporting Items for Systematic reviews and Meta-analysis guidelines (supplementary material S1) [25]. Searches on the electronic databases MEDLINE, Embase, the Cumulative Index to Nursing and Allied Health, the Allied and Complementary Medicine Database and the Cochrane Library were completed from January 2011 until August 2025. The search term “cystic fibrosis” was coupled with key terms including “body image” and “physical appearance” to retrieve relevant search results. Reference lists of included studies and relevant review articles were hand searched. Supplementary material S2 displays an example of the full search strategy. Search results were exported to EndNote 21 (Camelot UK Bidco Limited, UK) reference manager, and underwent duplicate removal, and Rayyan, an online software for systematic reviews that supports collaborative titles and abstract screening [26], was employed to establish article inclusion. The review was initially registered with the PROSPERO international prospective register of systematic reviews (www.crd.york.ac.uk/PROSPERO/ identifier number CRD42021286191).

### Eligibility criteria

Papers retrieved must not have been included in the previous critical review on body image published in 2012 [23], and therefore studies prior to January 2011 were not included. Research questions were written in the population, intervention, comparison, outcome format. Both experimental and observational study designs were included. Titles and abstracts were screened in Rayyan against the inclusion criteria. Thereafter, the authors screened full articles (C. Greaney, N. Cichy, G. O'Sullivan).

#### Inclusion criteria

Full-text studies examining young people and adults diagnosed with CF, defined as aged ≥16 years. It was a requirement for studies included to have investigated body image in young people and/or adults with CF in any setting. Qualitative and mixed-method studies were eligible for inclusion, assuming qualitative data could be clearly extracted. Quantitative data must have included measures of body image, while it was a requirement that qualitative data included discussions on body image (primary outcomes of interest). Secondary outcome measures (*e.g.* lung function; anthropometry), were presented if provided by studies but were not a requirement for inclusion. Comparators or control groups were not necessary but were included where available in observational studies.

#### Exclusion criteria

Nonhuman studies; paediatric studies; non-English language studies; articles not reporting primary outcomes of interest; data on people living with CF combined with data from individuals with other long-term conditions; conference abstracts.

### Extraction and synthesis

All articles meeting the inclusion criteria underwent data extraction (C. Greaney, N. Cichy, G. O'Sullivan). Information regarding the year, country or region, study design, population demographics, assessment methods, and the primary and secondary outcomes were extracted. Where values are provided, standard deviation and/or interquartile range (IQR) were reported, with mean values represented by mean±sd, and median values by median (IQR). Due to the heterogeneity in study design a meta-analysis was not completed. A mixed-methods review was completed with qualitative research represented narratively.

### Grading quality of studies and certainty of evidence

Risk of bias was examined using the Mixed Methods Appraisal Tool (MMAT), which separates criteria according to each study's design, with each criterion scored as “yes”, “no” or “unable to tell” (supplementary material S2) [27].

## Results

### Study selection and characteristics

**FIGURE 1:**
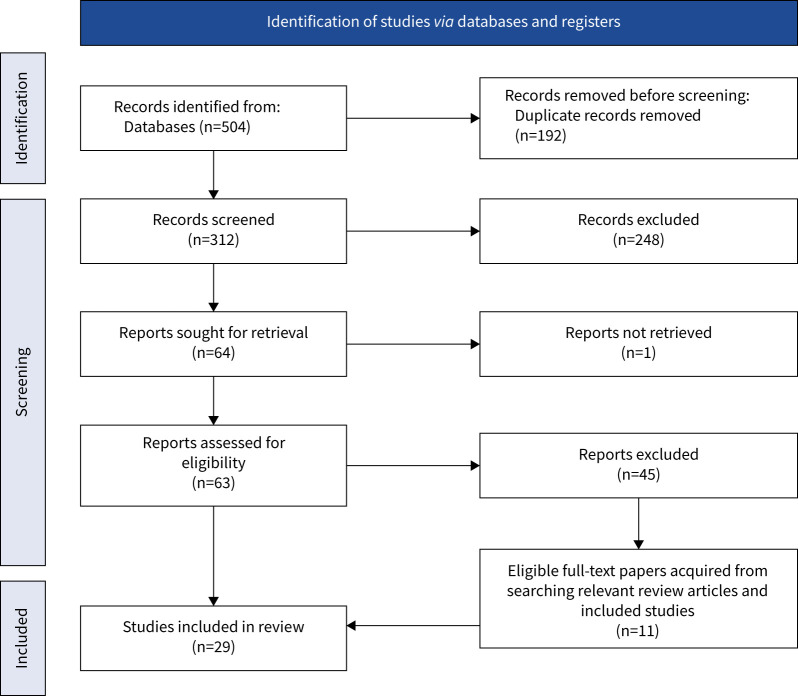
Preferred Reporting Items for Systematic Reviews and Meta-Analyses flow diagram showing search results.

Database searches yielded 504 research articles since January 2011. After removing duplicates (n=192), 312 records remained for title and abstract screening, of which 248 were excluded. Of the remaining 64 records, we were unable to retrieve one article, as its full text was not open access. The full texts of the remaining 63 records were accessed and reviewed for evaluation against inclusion criteria; 18 of which were eligible to be included in the final systematic review. Hand-searching reference lists of included studies and relevant review articles retrieved an additional 11 records (figure 1).

Studies were conducted from the years 2011–2025 in North America [28, 29] or the USA [30–35], the United Kingdom [36–41] or England [42], Ireland [43], Poland [44, 45], Denmark [46, 47], Spain [48, 49], France [50], Greece [51], Germany [52], Hungary [53], Brazil [54] and South Africa [55], with one multinational study [56]. Study designs included cross-sectional [29, 39, 41, 43–45, 47–54], prospective cohort/longitudinal [28, 31, 34, 35, 40, 42, 48, 50], randomised controlled trial (RCT) [36, 37, 46, 56], quasi-experimental [55], mixed-method (sequential quantitative–qualitative) [33] and qualitative [30, 38] studies. The sample size of the included studies ranges from nine to 631, representing n*=*3314 people living with CF, of whom >1868 were male participants, with one study (n=11) not describing gender distributions. Quantitative studies included addressed the topic of body image in an overall broader exploration of health-related quality of life (HRQoL) *via* the use of validated CF-specific instruments Cystic Fibrosis Questionnaire – Revised (CFQ-R) [57] and Cystic Fibrosis Quality of Life Questionnaire (CFQoL) [58], with higher body image domain scores for each representing better self-reported body image (0–100). Study characteristics are presented in table 1.

**TABLE 1:** Study characteristics

First author, year [ref.]	Country/region	Study design	Body image measure	PwCF	Study aim	Participant characteristics
**Dury, 2023 [50]**	France	Quantitative, cross-sectional, prospective pilot	CFQ-R	40	To assess the relationships between BMD and QoL	Male 27 (68) Age (years) 25 (12)^#^ BMI (kg·m^−2^) 21 (4)^#^ FEV_1_ (% pred) 54 (56)^#^ PI 31 (78) CFRD 12 (30) F508del homozygous 15 (38); heterozygous 22 (55); other 3 (8) Low BMD 10 (25) Normal BMD 30 (75)
**Tervo, 2023 [31]**	USA	Quantitative, prospective cohort	CFQ-R	41	To understand whether ETI therapy facilitates improvement of chronic rhinosinusitis symptoms, improves patient-perceived body image and eating behaviour, and alleviates olfactory impairment in PwCF To understand whether self-reported sense of smell improves after starting ETI and whether subjective improvement in olfaction is an independent mediator of improved BMI and self-reported QoL	Male: 13 (31.7) Age (years): 35.3
**Wildman, 2022 [36]**	UK	Quantitative, 12-month RCT	CFQ-R	607	To investigate the effectiveness of a multicomponent self-management intervention compared with usual care in adults with CF using pulmonary exacerbation incidence rate as the primary outcome	Usual care 303 Male 149 (49.2) Age (years) 30.3±10.8 Baseline BMI (kg·m^−2^) 22.5±4.2 Baseline FEV_1_ (% pred) 58.3±22.6 Intervention group 304 Male 148 (48.7) Age (years) 31.1±10.6 Baseline BMI (kg·m^−2^) 22.7±4.2 Baseline FEV_1_ (% pred) 60.7±23.5
**Friedman, 2022 [37]**	UK	Feasibility/acceptability pilot	CFQ-R	13	To pilot and assess the feasibility and acceptability of a CF-specific cognitive-behavioural preventive intervention for adults integrated into team-based care	Male 1 (7) Age (years) 28±7 FEV_1_ (% pred) 67±27
**Barrett, 2022 [38]**	UK	Qualitative	Semi-structured interview *via* telephone	9	To explore the perceptions and attitudes of food and food experience of adults with CF, how CF influences eating habits and relationship with food, and the practicalities of managing the complex dietary needs of a chronic life-limiting illness. It aims to contribute to the body of knowledge on nutritional management of CF from the patient's perspective	Male 6 (66.7) Age (years) 23–48 BMI (kg·m^−2^) 20.2–25.1 FEV_1_ (% pred) 27–98 PI 100% CFRD 66.7%
**Bray, 2021 [33]**	USA	Sequential quantitative–qualitative mixed methods	CFQ-R and semi-structured interviews	123	To explore gender differences in HRQoL and understand gender-specific facilitators and barriers to HRQoL	Male 57 (46) Age (years) 31.9±11.6 BMI (kg·m^−2^) 23.5±4.9 FEV_1_ (% pred) 61.0±23.3
**DiMango, 2021 [34]**	USA	Quantitative, prospective cohort study	CFQ-R	43	To measure the effect of ETI on patient-reported sinonasal and overall QoL, and to determine the relationship between changes in these two outcome measures	Male 14 (33) Age (years) 34.0 (30.5–37.5)^¶^ BMI (kg·m^−2^) 21.8 (21.0–22.6)^¶^ FEV_1_ (% pred) 63.5 (51.0–76.0)^¶^
**Flewelling, 2019 [32]**	USA	Quantitative, cross-sectional	CFQ-R	250	To examine the self-report of a large panel of adults with CF with regard to social support and examine the relationship between social support and health outcomes	Male 78 (39) Age (years) 34.7±10.2 (range 20–65)
**Cronly, 2019 [43]**	Ireland	Quantitative, cross-sectional	CFQ-R	147	To explore the associations of positive mental health, wellbeing, physical health and HRQoL in adults with CF	Male 64 (44) Age (years) 30.5±9.1
**Íscar-Urrutia, 2018 [48]**	Spain	Descriptive, prospective, cross-sectional	CFQ-R	23	To assess subjective sleep quality using validated questionnaires and objective sleep quality using polysomnography and to explore the relationship between sleep disturbances and HRQoL and its relationship to lung function	Male 9 (39) Age (years) 32±18 FEV_1_ (% pred) 69±22 PI 18 (78.2) CFRD 5 (21.7) F508del 79%; homozygous 30.4%; other mutation 17.4%
**Knudsen, 2017 [46]**	Denmark	Quantitative, feasibility RCT	CFQ-R	38	To establish the feasibility of a coaching intervention for young adults with CF	Male 11 (28.9) Age (years) 23.7 BMI (kg·m^−2^) 22.1 (3.8) FEV_1_ (% pred) 73.4±23.0 CFTR mutation classes 1 or 2: 36 (95) HbA1c (%): 6.2±1.5% Intervention 18 Control 20
**Helms, 2017 [30]**	USA	Qualitative	Semi-structured interviews and one closed-end question	20	To understand what the various components of a successful communication around body image between a patient and a provider would entail	Male 10 (50) Age (years) 17.5 BMI (kg·m^−2^) 19.4±2.5 FEV_1_ (% pred) 76.2±22.5 F508del homozygous 20%; heterozygous 5%; other 15%, unknown 60%
**Stofa, 2016 [51]**	Greece	Quantitative cross-sectional observational	CFQoL	77	To explore the QoL in CF adults in Greece	Male 34 (44.2) Age categories: 18–23 years: 22 (28.7); 24–28 years: 18 (23.3); 29–33 years: 18 (22.3); >33 years: 19 (24.7) Education level: none: 4 (5.2); primary: 6 (7.8); secondary 26 (33.8); tertiary: 41 (53.2)
**Knudsen, 2016 [47]**	Denmark	Quantitative, cross-sectional study	CFQ-R	67	To examine rates of adherence to prescribed treatments, assess symptoms of depression, evaluate HRQoL, and test the associations between adherence, depression and HRQoL in young adults with CF	Male 29 (43.3) Age (years) 24.1 (18–30) BMI (kg·m^−2^) 21.8±3.6 FEV_1_ (% pred) 72.2±23.0 CFRD 18 (28)
**Olveira, 2016 [49]**	Spain	Quantitative, cross-sectional	CFQ-R	336	To measure symptoms of depression and anxiety in a large sample of Spanish adult patients with CF and evaluate their relationship to demographics, health status and HRQoL	Male 174 (51.8) Age (years) 28.1±8.2 BMI (kg·m^−2^) 21.8±3.3 FEV_1_ categories: 35% pred 39 (11.6); 35–50% pred 70 (20.7); 50–80% pred 133 (39.6); >80% pred 94 (28) PI 244 (72.6) CFRD 70 (20.9)
**Tóth, 2016 [53]**	Hungary	Quantitative, cross-sectional	CFQ-R	57	To assess the QoL of adult PwCF in Hungary	Male 26 (45.6) Age (years) 28.3±9.0 BMI (kg·m^−2^) 19.4±2.5 FEV_1_ (% pred) 54.0±24.6
**Borowitz, 2016 [56]**	International	Quantitative, double-blind placebo-controlled RCT	CFQ-R	108	To explore in greater depth the improvements in weight and BMI after 48 weeks of treatment in the phase 3 trials of ivacaftor	Male 52 (48.1) Age (years) 30.0±8.2 FEV_1_ (% pred) 59.8 (15.2) BMI (kg·m^−2^) 22.9±3.6
**Shaw, 2016 [55]**	South Africa	Quantitative, quasi-experimental design	CFQ-R	11	To examine the effect of resistance training during nebulisation on spirometry, anthropometry, chest wall excursion, respiratory muscle strength and HRQoL	Baseline Resistance training group: 7 Body mass (kg) 53.8±13.2 FEV_1_ (% pred) 58.3±13.0 Non-exercising control group: 4 Body mass (kg) 64.3±30.7 FEV_1_ (% pred) 67.2±10.4
**Forte, 2015 [54]**	Brazil	Quantitative, cross-sectional	CFQoL	51	To explore and evaluate the association between clinical, lung function, sleep quality, and polysomnographic variables in young CF adults	Male 27 (52.9) Age (years) 25.1 (8.8) BMI 20.5±2.4 FEV_1_ (% pred) 57.7±24.7
**Uchmanowicz, 2015 [44]**	Poland	Quantitative, cross-sectional observational	CFQoL	30	To evaluate the QoL of individuals with mucoviscidosis and to explore how QoL assessments vary based on age and sex	Male 11 (36.7) Age (years) 24.8±7.0
**Dębska, 2014 [45]**	Poland	Quantitative, cross-sectional	CFQoL	45	To analyse the QoL in CF patients depending on the severity of the disease and methods of its treatment	Age (years) ≥18
**Targett, 2014 [39]**	UK	Quantitative, observational cross-sectional	CFQ-R	254	To investigate and explore the associated factors in adults with CF and employment status	Male 137 (54) Age (years) 26 (21–34)^#^ BMI (kg·m^−2^) 22.4 (15–41)^¶^ FEV_1_ (% pred) 60 (12–136)^¶^ CFRD 85 (34) Employed 127 Unemployed 76
**Dill, 2013 [35]**	USA	Quantitative, prospective longitudinal panel	CFQ-R	333	To explore the trends in HRQoL in adults with CF	Male 150 (45) Age (years) 32.5±10.7 FEV_1_ (% pred) 59.8±22.4 PI 78% CFRD 15%
**Bradley, 2013 [40]**	UK	Quantitative, prospective cohort	CFQ-R	94	To explore health status associated with pulmonary exacerbations in patients with CF and chronic *P. aeruginosa* infection	Male 48 (51.1) Age (years) 28.5±8.2 FEV_1_ (% pred) 58.7±26.8
**Ashish, 2012 [42]**	England	Quantitative, cohort study	CFQ-R	157	To assess the impact on HRQoL in adult CF patients of chronic infection with the LES of *P. aeruginosa*	LES *P. aeruginosa* strain 93 Male 55 (59) Age (years) 26 (26–31)^#^ BMI (kg·m^−2^) 21.7±3.5 FEV_1_ (% pred) 65±23 *P. aeruginosa* strain (other) 44 Male 28 (63.6) Age (years) 22 (20–28)^#^ BMI (kg·m^−2^) 22.6±3.6 FEV_1_ (% pred) 69±23 No *P. aeruginosa* infection 20 Male 11 (55) Age (years) 21 (19–29)^#^ BMI (kg·m^−2^) 25±4.8 FEV_1_ (% pred) 77.8±26
**Bouka, 2012 [52]**	Germany	Quantitative, cross-sectional	CFQ-R	55	To investigate the association between daytime sleepiness and sleep quality in clinically stable adult CF outpatients	Male 30 (54.5) Age (years) 34.4±7.5 BMI (kg·m^−2^) 21.5±2.9 FEV_1_ (% pred) 59.0±23.6
**Yohannes, 2012 [41]**	UK	Quantitative, cross-sectional, observational	CFQoL	121	To explore the prevalence and factors associated with anxiety and depression, including QoL, in a large cohort of adults with CF	Male 65 (58.5%) Age (years) 30±8.8 BMI (kg·m^−2^) 22.0±2.6 FEV_1_ (% pred) 47±11.2 PI 39 (32) CFRD 50 (41)
**Sawicki, 2011 [28]**	North America	Quantitative, longitudinal prospective observational cohort	CFQ-R	631	To examine the relationship between changes in health status and changes in CFQ-R scores outside the context of a clinical trial	Age (years) 26.9±9.7 Male 328 (52) FEV_1_ (% pred) 61±23
**Sawicki, 2011 [29]**	North America	Quantitative cross-sectional	CFQ-R	199	To examine the relationship between illness perception, health status, and HRQoL in a cohort of adults with CF	Male 76 (38) Age (years) 35.8±10.3 BMI (kg·m^−2^) 22.2±4.0 FEV_1_ (% pred) 62.5±22.8

Data are presented as n, mean±sd, n (%) or range, unless otherwise stated. PwCF: people living with cystic fibrosis; CFQ-R Cystic Fibrosis Questionnaire – Revised; BMD bone mineral density; BMI: body mass index; FEV_1_: forced expiratory volume in 1 s; PI: pancreatic insufficiency; CFRD: cystic fibrosis-related diabetes; USA: United States of America; ETI elexacaftor/tezacaftor/ivacaftor; UK: United Kingdom; RCT: randomised controlled trial; CF: cystic fibrosis; CFTR: cystic fibrosis transmembrane conductance regulator; HRQoL: health-related quality of life; QoL: quality of life; CFQoL: Cystic Fibrosis Quality of Life Questionnaire; LES: Liverpool Epidemic Strain; *P. aeruginosa*: *Pseudomonas aeruginosa*. ^#^: median (interquartile range); ^¶^: mean (95% CI).

Incidences of pancreatic insufficiency were published in six studies, with most reporting incidences between 70% and 100% [35, 38, 48–50], and one study reporting 32% [41]. Differences in body image could not be assessed between pancreatic-insufficient and pancreatic-sufficient individuals in this review, as no data on pancreatic sufficient participants were provided. Incidence of CF-related diabetes was reported in eight studies ranging from 15% [35] to 67% [38]. The most frequently reported CF mutation was F508del. BMI, forced expiratory volume in 1 s (FEV_1_ % pred) and further demographic data are presented in table 1.

Both qualitative studies and the mixed-methods study employed semi-structured interviews to explore body image and body image-related topics [30, 33, 38]. While Barrett *et al.* [38] did not ask any semi-structured interview questions specifically related to body image, “balancing health and body image goals” arose as a key theme in the study. The second qualitative study interviewed both people living with CF and providers; however, it was possible to extract data on just people living with CF [30]. The primary focus of this qualitative study was “to understand what the various components of a successful communication around body image between a patient and a provider would entail”. The study included participants as young as 15 years [30]. Despite the minimum age of 16 years being applied to our inclusion criteria for this review, it was deemed appropriate to include this study, as most participants met inclusion criteria, with a mean age of 17.5 years being reported. In the mixed-methods study by Bray *et al.* [33] the goal of the semi-structured interviews was to explore gender differences in HRQoL and understand gender-specific facilitators and barriers to HRQoL, which discussions related to body image contributed to.

### Quantitative assessment of body image in CF

Quantitative data on CFQ-R/CFQoL body image domain scores are reported in table 2. There were variations in the parameters which reported data across studies (*e.g.* mean±sd, median (IQR)), which restricts a direct comparative review analysis in this review. A CFQ-R body image domain score was reported across 16 quantitative studies, with scores for total cohorts ranging from 58±19 to 78±23. In the context of time periods within CF, pre-modulator era (pre-2012) CFQ-R body image domain scores range from 64±27 to 70±27; scores within the initial rollout period (2012–2019) ranged from 58±19 to 81 (post 48-week ivacaftor intervention), and in the era of widespread modulator use (2020–present), scores range from 63±32 to 67±31. A further five studies reported a CFQoL body image domain score, with scores ranging from as low as 21±26 (people living with CF with no education) to as high as 76. CFQoL body image domain scores were not reported in the era of widespread modulator use.

**TABLE 2:** Quantitative data on body image in adults and young people living with cystic fibrosis (CF)

First author, year [ref.]	Body image domain scores (0–100)^#^		Effect size	r	R^2^	p-value	Comments
	Baseline	Post-intervention					
**Widespread use of modulators (2020–present)**							
Dury, 2023 [50]	67 (31)						
Low/normal BMD	67 (34)/67 (22)					>0.050	
Tervo, 2023 [31]							
After 3 months ETI treatment						0.777^¶^	
Wildman, 2022 [36]							
Usual care/multicomponent self-management intervention	66±29/66±28	1.7 (−1.4–4.8)^+^	0.06				
Friedman, 2022 [37]							
After eight sessions CF-specific cognitive behaviour therapy			−0.22				
Bray, 2021 [33]	63±32						
Gender (male/female)							0.79
**Initial rollout phase of modulators (2012–2019)**							
DiMango, 2021 [34]							
After 3 months ETI treatment	Mean (95% CI) 75.5 (70–82)	Mean (95% CI) 77.8 (72–84)				>0.05	
Flewelling, 2019 [32]	65 (27)						
Perceived social support (dependent)					0.31^§^	<0.001^¶^	β: 0.63
Cronly, 2019 [43]							
Age, gender, BMI, FEV_1_%, WEMWBS					0.39	<0.001^¶^	β: gender=0.14, p=0.038; WEMWBS=0.80, p<0.001 Age, BMI and FEV_1_%: p>0.05
Íscar-Urrutia, 2018 [48]	58±19						
Duration of sleep						0.071	
Perceived sleep efficiency				−0.520^ƒ^		0.016	
Knudsen, 2017 [46]							
Control/coaching intervention	76±26/74±25	86±16/69±27^##^	0.91, 0.74^¶¶^			0.001, 0.047^¶¶^	
Stofa, 2016 [51]							
Gender (male/female)	31±30/43±35^++^					0.118^§§^	
Age (18–23/24–28/29–33/>33 years)	41±38/29±23/24±30/50±33^++^					0.078^§§^	
Education (none or primary/secondary/tertiary)	21±26/28±30/46±34					0.025	
Knudsen, 2016 [47]	78±23						
Depression/no depression	61±28/85±17		−1.12			<0.001	
Work or education ability/disability	81±21/56±28		−1.18			0.002	
Olveira, 2016 [49]							
Depression (HADS-D ≥8)/normal (HADS-D <8)	51±21/79±17					<0.001	Significant differences between: HADS-D depression/FEV_1_ <50% and HADS-D normal/FEV_1_ <50% HADS-D depression/FEV_1_ ≥50% and HADS-D normal/FEV_1_ ≥50 HADS-D normal/FEV_1_ ≥50% and HADS-D depression/FEV_1_ <50% HADS-D depression/FEV_1_ ≥50% and HADS-D normal/FEV_1_ <50%
Anxiety (HADS-A ≥8)/normal (HADS-A <8)	60±21/82±16					<0.001	Significant differences between: HADS-A anxiety/FEV_1_ <50% and HADS-A normal/FEV_1_ <50% HADS-A anxiety/FEV_1_ ≥50% and HADS-A normal/FEV_1_ ≥50% HADS-A normal/FEV_1_ ≥50% and HADS-A anxiety/FEV_1_ <50% HADS-A anxiety/FEV_1_ ≥50% and HADS-A normal/FEV_1_ <50%
Tóth, 2016 [53]	54±28						
BMI				0.656^ƒƒ^		<0.01	
Body fat percentage				0.433^ƒƒ^		<0.01	
Borowitz, 2016 [56]							
Placebo group	79	75				0.0194^###^	
Ivacaftor group	78	81					
Shaw, 2016 [55]							
Resistance training group	65±26	46±24				0.046	
Control group	67±24	42±11				0.036	
Forte, 2015 [54]	76^++^				0.23	<0.05^¶^	β: gender (male)=0.339; BMI=0.298
**Pre-modulator era (pre-2012)**							
Uchmanowicz, 2015 [44]							
Gender (male/female)	47/49^++^					0.82	
Age (≤25/>25 years)	49/45^++^					0.37	
Gender and age						>0.05	
Dębska, 2014 [45]							
Transplant status (post/awaiting/stable)	69/42/65^++^					0.008	
Targett, 2014 [39]	Mean (95% CI) 67 (64–71)						
Employed/unemployed	Mean (95% CI) 69 (64–74)/60 (54–66)					<0.05	
Dill, 2013 [35]	64±27	67±26^¶¶¶^				>0.05	
Age, gender, education, FEV_1_%, weight %, pancreatic status, number of exacerbations					0.19	<0.06^¶^	β: female=9.80, weight %=0.25, number of exacerbations=−1.56, p<0.001; FEV_1_%=0.10, pancreatic sufficiency=3.18, p<0.05; age, education, p>0.05; individual variability in body image domain: 5th percentile=−7.8; 95th percentile=8.1
Bradley, 2013 [40]							
EQ-5D utility index				0.203^ƒ^		<0.05	
Ashish, 2012 [42]							
Other/no *P. aeruginosa*	78 (45)/100 (0)					0.008	
LES/no *P. aeruginosa*	67 (56)/–					<0.001	
LES/other *P. aeruginosa*						>0.05	
Bouka, 2012 [52]							
Pittsburgh Sleep Quality Index (dependent)						0.086^¶,+++^	
Yohannes, 2012 [41]	70±21^++^						
Depression						<0.001	
Anxiety						<0.001	
Sawicki, 2011 [28]	70±27	0.7±19.0^§§§^				>0.05	
Change in weight, FEV_1_%, MSSA status					0.047^ƒƒƒ^	<0.05	β (95% CI): change in weight:=8.16 (4.17–12.17); change in FEV_1_=−0.23 (−0.42–−0.05); MSSA status=−6.33 (−12.66–0.00)
Sawicki, 2011 [29]							
Age, gender, FEV_1_%, BMI, exacerbations, *B. cepacia* positive, *P. aeruginosa* positive					0.24^####^	<0.05^¶^	β: female=17.7, BMI=2.0 kg·m^−2^, ≥1 exacerbations=−8.4, p<0.05; age, FEV_1_%, *B. cepacia* positive, *P. aeruginosa* positive, p>0.05
MSAS respiratory, gastrointestinal, and psychological symptoms					0.14^####^	<0.05^¶^	β: MSAS gastrointestinal symptoms=−10.8; MSAS psychological symptoms=−5.8, p<0.05; MSAS respiratory symptoms, p>0.05
IPQ-R illness consequences, illness, coherence, illness timeline – cyclical, personal control, treatment control					0.40^####^	<0.05^¶^	β: IPQ-R illness consequences=−6.6, p<0.05; IPQ-R illness coherence, IPQ-R illness timeline – cyclical, IPQ-R personal control, p>0.05

Data are presented as median (interquartile range) or mean±sd, unless otherwise stated. BMD: bone mineral density; ETI: elexacaftor/tezacaftor/ivacaftor; BMI: body mass index; FEV_1_: forced expiratory volume in 1 s; WEMWBS: Warwick–Edinburgh Mental Wellbeing Scale; HADS-D/A: Hospital Anxiety and Depression Scale – Depression/Anxiety; EQ-5D: EuroQol 5-dimension; *P. aeruginosa*: *Pseudomonas aeruginosa*; LES: Liverpool Epidemic Strain; MSSA: methicillin-susceptible *Staphylococcus aureus*; *B. cepacia*: *Burkholderia cepacia*; MSAS: Memorial Symptom Assessment Scale; IPQ-R: Illness Perception Questionnaire – Revised. **^#^**: scores were generated from the Cystic Fibrosis Questionnaire – Revised, unless otherwise stated; ^¶^: linear regression model p-value; ^+^: adjusted difference in means (95% CI) at 12-month follow up. All other analyses adjusted for past year intravenous days, centre, and outcome measure at baseline; ^§^: adjusted for weight percentile, diagnosis of diabetes, pancreatic sufficiency status, and colonisation with *B. cepacia, S. aureus* and *P. aeruginosa*; ^ƒ^: Pearson's r correlation p-value; ^##^: value provided is at 1-year follow-up (post-intervention) point. Midway point (5 months): 84±20/75±19; 11 months (post-intervention): 80±21/73±26; ^¶¶^: first value is group factor, second value is time factor; ^++^: Cystic Fibrosis Quality of Life Questionnaire; ^§§^: ANOVA F-test; ^ƒƒ^: Spearman's rank correlation coefficient (r) p-value; ^###^: treatment difference from baseline to week 48: 7.6 (95% CI 1.3–14.0); ^¶¶¶^: value represents 21-month point; 3 months=67±25, 6 months=64±24, 9 months=66±25, 13 months=68±26, 18 months=67±26; ^+++^: adjusted for age, gender, BMI, ratio of the volume of air exhaled to the total volume of air that can be exhaled, and *Pseudomonas* status; ^§§§^: change (mean±sd) after 9–15 months; ^ƒƒƒ^: adjusted for gender and age; ^####^: adjusted for predicted survivorship score.

#### Gender

While in the pre-modulator era, linear regression models from one longitudinal [35] and one cross-sectional study [29] identified female gender as a significant positive predictor of better body image domain scores (β=9.80, p<0.001 [35]; β=17.7, p<0.05 [29]), research in the initial rollout phase of modulators were conflicting, with both male (β=0.339, p<0.05) [54] and female (β=0.14, p=0.038) [43] gender identified as significant predictors of body image domain scores. A further four studies displayed no significant differences between genders when conducting nonpredictive testing [33, 44, 51].

#### Body composition and bone density

While two cross-sectional studies reported BMI as a positive predictor of better body image domain scores (β=2.0, p<0.05 [29]; β=0.298, p<0.05 [54]), and moderate correlations were observed between body image domain scores and BMI (r=0.656, p<0.01) and body fat percentage (r=0.433, p<0.01) in Tóth *et al.* [53], a more recent cross-sectional study observed no predictive relationship between BMI and body image domain scores [43]. Pre-modulator era research also reported that an individual's weight (β=0.25, p<0.001) [35] and change in weight (β=8.16, p<0.05) [28] were predictors of body image domain scores. No significant differences were reported in body image domain scores between those with normal and low bone mineral density [50].

#### Psychology

The Memorial Symptom Assessment Scale (MSAS) Psychological Symptoms (β=−5.8, p<0.05) [28] and Warwick–Edinburgh Mental Wellbeing Scale (β=0.80, p<0.001) [43] were significant predictors of body image domain scores. Anxiety and depression were associated with body image domain scores in a cross-sectional study within the pre-modulator era (p<0.001) [41] and in a study from the initial rollout out phase of modulators that used the Hospital Anxiety and Depression Scale (HADS) (HADS-Depression p<0.001; HADS-Anxiety p<0.001) [49]. The CFQ-R body image domain score was observed as being a significant predictor of perceived social support (β=0.63, p<0.001).

#### CF-specific therapies, treatments and interventions

A double-blinded placebo-controlled RCT displayed significant improvement in CFQ-R body image domain scores following 48 weeks of ivacaftor treatment (treatment difference 7.6, 95% CI 1.3–14.0; p*=*0.0194). However, in two prospective cohort studies no change in CFQ-R body image domain scores were observed following 3-month elexacaftor/tezacaftor/ivacaftor (ETI) treatment (p=0.777 [31]; p>0.05 [34]), and one cross-sectional study reported no associations between modulator use and CFQ-R body image domain scores. Dębska *et al.* [45] reported a significant difference between transplant status, with those awaiting transplant reporting the worst CFQoL body image domain scores (post-transplant 69, awaiting transplant 42, stable 65; p=0.008). A quasi-experimental study reported CFQ-R body image domain scores worsened significantly in both a resistance-trained intervention group (p=0.046), and in the control group of the study (p=0.036). The authors illustrated that this finding was not unexpected in the resistance-trained group as no improvements in body composition were demonstrated following the intervention [55]. A feasibility RCT reported significant improvement to CFQ-R body image domain scores following the administration of life coaching (up to 10 coaching sessions (60–90 min long) over 6–9 months, with both study group (Cohen's d=0.91, p=0.001) and time (Cohen's d=0.74, p=0.047) significant factors [46]. A 12-month RCT investigating the impact of a multicomponent self-management intervention *versus* usual care reported an adjusted difference in the mean CFQ-R body image domain score of 1.7 (95% CI −1.4–4.8; Cohen's d=0.06). A feasibility pilot study reported that eight sessions of CF-specific cognitive behaviour therapy had a small negative effect size (Cohen's d=−0.22) on CFQ-R body image domain scores [37].

#### Demographic and clinical parameters

Mixed results are presented in relation to FEV_1_%, with pre-modulator era longitudinal studies reporting FEV_1_% (β=0.10, p<0.05) and exacerbation frequency (β=−1.56, p<0.001) [35] or a change in FEV_1_% (β=−0.23, p<0.05) [28] as predictive and cross-sectional studies from both the pre-modulator era and initial rollout phase observing no predictive relationship between FEV_1_% [29, 43] or MSAS respiratory [29] and body image domain scores. However, reports of one or more pulmonary exacerbations were observed to be a predictor in one of these cross-sectional studies (β=8.4, p<0.05) [29].

Associations were observed between employment (p<0.05) [39], work or education ability (p=0.002) [47] and education (none or primary/secondary/tertiary p=0.025) [51], and body image domain scores; however, a longitudinal study reported no predictive relationship between education and CFQ-R body image domain scores [35].

One study reported pancreatic sufficiency as a predictor of body image domain scores (β=3.18, p<0.05) [35].

Age was not observed as predictive or associated to body image domain scores in any study. Sleep quality and duration were not observed to be associated with body image domain scores [48], nor was body image domain score predictive of the Pittsburgh Sleep Quality Index [52]. However, sleep efficiency was reported as being significantly associated with CFQ-R body image domain scores (r*=*0.520, p=0.016) [48].

While being *Burkholderia cepacia* or *Pseudomonas aeruginosa* positive was not predictive of body image domain scores [29], a longitudinal study reported methicillin-susceptible *Staphylococcus aureus* status as predictive (β=−6.33, p<0.05) [28] and a cohort study reported significant differences in CFQ-R body image domain scores between those with the Liverpool Epidemic Strain of *P. aeruginosa versus* no *P. aeruginosa* (p<0.001) and those with another *P. aeruginosa* strain *versus* no *P. aeruginosa* (p*=*0.008) [42].

### Qualitative reporting of body image in CF

All qualitative studies followed the thematic analysis approach developed by Braun and Clarke [59] to analyse semi-structured interview transcripts, with the authors of each subsequentially developing key themes (table 3) [30, 33, 38]. Participants across studies consistently acknowledged body image as an important yet under-discussed aspect of living with CF, with positive rapport with healthcare providers described as something that would enhance openness in discussing sensitive topics [30, 33, 38]. Even so, participants in both Barrett *et al.* [38] and Helms *et al.* [30] acknowledged uncertainty with which healthcare professional should have the conversation with them about body image and how conversations should be approached.

**TABLE 3:** Key themes from qualitative and mixed-methods studies on body image in adults and young people living with cystic fibrosis (CF)

First author, year [ref.]	Key themes	Illustrative focus
**Barrett, 2022** [38]	1) Sustained influence of eating experiences in childhood 2) Eating for health: weight gain to prevent infection 3) Balancing health and body image goals 4) I'm different 5) Strategies for managing food intake 6) Support from family, friends and the CF team	Childhood struggles with food shaped adult self-perceptions of body and eating; food viewed as treatment reinforced functional over aesthetic views of the body; tension between looking “well” and feeling healthy; enjoying being unrestricted eaters contrasted with restrained eating to manage weight/image; family/CF team involvement influenced body image positively (encouragement) and negatively (perceived pressure)
**Bray, 2021** [33]	1) Biological and physiological factors 2) Functional status 3) Perceived symptom status 4) External factors 5) Personal perception	Gender ideals shaped body image (male concerns of thinness *versus* muscularity, female concerns with bloating/weight gain); functional limitations, coughing and equipment use undermined appearance and confidence; social media a double-edged sword, being both supportive, but also reinforced comparisons affecting body image; external factors indirectly shaped body image, while fertility concerns, anxiety, and depression deepened body dissatisfaction
**Helms, 2017** [30]	1) Routine part of care 2) Support and comfort with communication 3) Supporting psychosocial health separate from physical health 4) Uncertainty and variability of approaches	Body image rarely discussed in clinic; participants wanted providers to initiate conversations on “touchy subjects”; empathetic communication enhanced openness, while weight-focused dialogue (*e.g.* tube feeding threats) worsened body image; uncertainty about who should address body image and how it should be approached reflected unease with the topic in care

Barrett *et al.* [38] highlighted the lasting influence of childhood eating experiences, where poor appetite, food aversions, and tube-feeding threats shaped later relationships with food. While some enjoyed the freedom of high-calorie diets in adulthood, eating was often perceived as treatment rather than pleasure, with many describing eating lots of food to maintain a healthy weight as a priority for preventing chest infections and for their wellbeing, which did not always coincide with appearance preferences. Some participants restricted intake to influence weight or protect dental health, while others expressed pride in eating without restriction. Family and CF team support was valued, but sometimes experienced as pressure when expectations around food intake differed [38].

Bray *et al.* [33] highlighted gender differences in body image perceptions. Men associated thinness with weakness, aspiring to be muscular, while women desired thinness and described distress linked to bloating and modulator-related weight gain. Physical limitations restricted activities, while visible signs of illness, such as persistent coughing or reliance on medical equipment, undermined self-image, with social media described as a “double-edged sword”, acting as both a supportive and anxiety-inducing influence. External factors, including finances, family support, and healthcare relationships, indirectly shaped body image. Mental health, fertility concerns, and personal outlook further influenced how participants perceived their bodies, with a positive outlook described as empowering and protective, while anxiety and depression were linked to illness progression and physical changes [33].

In contrast to Barrett *et al.* [38], participants in Helms *et al.* [30] described a freedom of being able to eat unrestrictedly. However, participants were reluctant to initiate conversations around body image and had a preference for clinicians to raise the topic as part of routine care. Participants also portrayed discomfort when body image was framed only in terms of weight gain or feeding tube threats, perceiving providers as focused on weight rather than holistic wellbeing. Communication style was described as pivotal, with empathetic, authentic engagement highlighted as encouraging openness, while dismissiveness discouraged disclosure. Participants emphasised the need to address psychosocial health separately from medical targets [30]. Alongside the semi-structured interviews conducted in Helms *et al.* [30], one closed-ended question about the frequency of body image discussions was distributed, reporting that 85% (n*=*17) of CF participants had never had a conversation with a healthcare provider about their body image or how they felt about their bodies.

### Quality assessment and risk of bias

All studies were assessed using the MMAT [27], and this appraisal is evidenced in table 4. Quality appraisal indicated that all included studies met the initial screening criteria. The three studies with qualitative elements fulfilled all methodological criteria [30, 33, 38]. Among the three RCTs, one achieved full scores [56] while the others showed some uncertainty regarding blinding and outcome data [36, 46]. The majority of nonrandomised quantitative studies demonstrated generally good methodological quality, although several had unclear or negative ratings for confounding [32, 43, 53–55] and completeness of data [31, 33, 34, 37, 40, 44, 45, 48, 50, 51, 53, 55]. The single mixed-methods study met all criteria for the mixed-methods element of MMAT [33].

**TABLE 4:** Quality assessment of studies using the Mixed Methods Appraisal Tool [27]

First author, year [ref.]	Screening		Validity questions				
	S.1	S.2					
**Qualitative**			1.1	1.2	1.3	1.4	1.5
Barrett, 2022 [38]	+	+	+	+	+	+	+
Bray, 2021 [33]^#^	+	+	+	+	+	+	+
Helms, 2017 [30]	+	+	+	+	+	+	+
**Quantitative randomised controlled trials**			2.1	2.2	2.3	2.4	2.5
Wildman, 2022 [36]	+	+	+	+	?	?	+
Knudsen, 2017 [46]	+	+	+	+	?	−	+
Borowitz, 2016 [56]	+	+	+	+	+	+	+
**Quantitative nonrandomised**			3.1	3.2	3.3	3.4	3.5
Dury, 2023 [50]	+	+	?	+	+	−	+
Tervo, 2023 [31]	+	+	?	+	+	?	+
Friedman, 2022 [37]	+	+	?	+	+	−	+
DiMango, 2021 [34]	+	+	?	+	+	−	+
Flewelling, 2019 [32]	+	+	?	+	?	+	+
Cronly, 2019 [43]	+	+	+	+	?	+	+
Íscar-Urrutia, 2018 [48]	+	+	?	+	+	−	+
Stofa, 2016 [51]	+	+	?	+	+	?	+
Knudsen, 2016 [47]	+	+	+	+	+	+	+
Olveira, 2016 [49]	+	+	+	+	+	+	+
Tóth, 2016 [53]	+	+	?	+	?	?	+
Shaw, 2016 [55]	+	+	?	+	?	−	+
Forte, 2015 [54]	+	+	?	+	?	+	+
Uchmanowicz, 2015 [44]	+	+	?	+	+	?	+
Dębska, 2014 [45]	+	+	?	+	+	?	+
Targett, 2014 [39]	+	+	+	+	+	+	+
Dill, 2013 [35]	+	+	+	+	+	+	+
Bradley, 2013 [40]	+	+	+	+	+	?	+
Ashish, 2012 [42]	+	+	+	+	+	+	+
Bouka, 2012 [52]	+	+	+	+	+	+	+
Yohannes, 2012 [41]	+	+	+	+	+	+	+
Sawicki, 2011 [28]	+	+	+	+	+	+	+
Sawicki, 2011 [29]	+	+	+	+	+	+	+
Bray, 2021 [33]^#^	+	+	?	+	+	?	+
**Mixed methods**			5.1	5.2	5.3	5.4	5.5
Bray, 2021 [33]	+	+	+	+	+	+	+

+: answer to validity question was yes; ?: answer to validity question was unclear; −: answer to validity question was no. ^#^: mixed-methods study.

## Discussion

This is the first mixed-methods systematic review of body image in adults and young people with CF, showing that body image disturbances are prevalent, evolving, and clinically important in the modulator era.

### Quantitative reports of body image

This review highlights the complexity of assessing body image in people living with CF and the variability in findings across studies and treatment eras. CFQ-R and CFQoL body image scores generally fell in the low-to-moderate range, but variable reporting limited comparability. When contextualised within treatment advances there was evidence of improvement in the early modulator rollout period, but little consistent effect in the widespread modulator era.

Gender effects were inconsistent across eras, suggesting that with the shifting weight trajectories since the introduction of modulators, findings of the 2012 critical review that female people living with CF have better body image than male counterparts [23] may no longer hold true. Instead, other psychosocial or clinical factors appear to be more salient in shaping body image.

Body composition findings were also mixed. While earlier studies in this review reported higher BMI, weight and weight change as significant predictors of body image, a more recent study showed no consistent associations. This was replicated in Greaney *et al.* [60], whereby no significant differences in CFQ-R body image domain scores were reported with BMI, whether assessed continuously or by cut-offs. This variability suggests that although weight remains an important clinical and psychosocial factor in CF, its relationship with body image may be increasingly complex in the modulator era. A further challenge lies in the applicability of existing tools such as the CFQ-R and CFQoL. Current tools place more weighting on items that align with undernutrition (*e.g.* “I am too thin”), underrepresenting concerns about being overweight or obese in the modulator era. Given that body image disturbances are prevalent among overweight and obese individuals in the general population, and often persist even after weight loss [61], there is a growing imperative to support people living with CF in ways that reduce body image concerns and promote holistic health. This includes addressing the psychosocial factors linked to body image disturbances as well as the broader health challenges that can occur with changes in body composition.

Psychological wellbeing demonstrated more consistent associations. Higher anxiety and depression were strongly linked to poorer body image, while measures of wellbeing predicted better body image outcomes. Social support also appears to be important for body image, but is complicated by cross-infection risks that restrict peer connections in CF [62]. These barriers may contribute to social isolation, which is associated with adverse health outcomes in the general population [63], and within CF, poorer social functioning (CFQ-R domain) has been linked to reduced physical and mental health (n=188, R*^2^=*0.36, p<0.05) [29]. This highlights social support as a key factor to consider when forming interventions targeted at preventing and improving body image, as well as physical and mental health concerns among people living with CF.

Therapeutic interventions provided mixed results. A 48-week ivacaftor trial improved body image scores, yet ETI studies found no body image impact despite significant increases in BMI (p<0.0001 [31]; p<0.05 [34]). This may reflect short 3-month follow-ups, which may not allow sufficient time for weight-related changes to translate into measurable body image concerns, and questionnaires focused mainly on undernutrition. Other interventions, including life coaching, improved body image scores, while resistance training, cognitive behavioural therapy, and multicomponent self-management interventions had neutral or negative effects. These findings underscore the need for interventions that directly target psychosocial aspects of body image, rather than assuming improvements in physical health will translate automatically into positive body image outcomes.

The mixed associations between clinical and demographic parameters and body image reflect its experiential nature [18]. For people living with CF, body image relates not only to appearance, but also to functional abilities such as breathing, digestion and daily participation. Evidence linking pulmonary function, exacerbations, pancreatic status and income with body image highlights this interplay and is further evidenced by Greaney *et al.* [60] which found that median (IQR) body image scores differed by income (full-time income: 88.9 (22.2), part-time income: 55.6 (55.6), no income: 77.8 (33.3); p=0.003) and pancreatic sufficiency (insufficient: 77.8 (33.3), sufficient: 88.9 (22.2); p=0.041), although not by FEV_1_%. Similar patterns are seen in rheumatoid arthritis, where health and activity restrictions shaped body image and self-esteem [64, 65]. Thus, body image in CF is not an isolated construct, but one that is deeply intertwined with functional health, echoing patterns seen in other chronic conditions where the body's ability to sustain everyday life shapes self-perception. This underscores the need for clinical approaches that address both appearance and function-related aspects of body image when supporting people living with CF.

The quantitative findings emphasise the importance of incorporating routine body image screening into CF care, particularly using tools that are sensitive to both undernutrition and emerging overweight/obesity concerns in the modulator era. Clinicians should be aware that psychological wellbeing and social support are closely tied to body image and consider integrated psychosocial assessment as part of standard care. Future research should prioritise the development and validation of CF-specific body image assessment instruments that reflect contemporary treatment realities, alongside longitudinal and intervention studies that test strategies to prevent and address body image disturbances.

### Qualitative reports of body image

The qualitative findings provide important context to the quantitative results, emphasising how body image in CF is deeply embedded in lived experiences of eating, functioning and social interaction. The qualitative findings further emphasise the importance of appropriate social support when addressing body image among people living with CF. Across all three studies, participants highlighted that interpersonal connections and supportive communication shaped how comfortable they felt discussing sensitive issues. In Helms *et al.* [30], participants reflected that clinicians who showed genuine interest in their overall wellbeing made them more receptive to discussing “touchy subjects” such as body image. Despite this, only 15% of participants had ever discussed body image with a healthcare provider, reflecting an unmet need for structured support. Bray *et al.* [33] echoed this in their accounts of functional and social limitations, where positive rapport with healthcare providers and supportive family members enhanced openness and coping, whereas negative interactions reinforced discomfort in raising appearance- or function-related concerns.

The disconnect between perceived importance and clinical practice are replicated in a study of CF providers perspectives on assessing disordered eating and body image (n=232) [66]. Although most providers acknowledged body image as important and supported routine screening, few felt confident or screened regularly, citing limited knowledge as a barrier. Together, these findings highlight a gap between recognition of body image concerns and the confidence and training required to address them, reinforcing the need for a multidisciplinary approach where psychosocial specialists play a greater role.

The qualitative studies also highlight how body image in CF shifts across the life course. Barrett *et al.* [38] described transitioning from difficult childhood eating experiences, into adulthood, where the ability to consume high-calorie diets unrestrictedly was viewed positively, yet restrained eating and weight concerns persisted. Childhood reflections align with a meta-analysis which reported that children and adolescent people living with CF experience greater body image disturbances than peers (g=−0.50), similar to obesity and scoliosis [67]. Bray *et al.* [33] added further nuance, illustrating gender-specific experiences that align with general population body image concerns [22], with men often equating low weight with weakness and reduced masculinity, and women reporting dissatisfaction with bloating or weight gain, sometimes linked to CFTR modulator therapies. With 63% of providers in Kass *et al.* [66] reporting an impact of modulators on body image disturbances, this indicates that despite clinical improvements, concerns related to body weight, shape, and function remain relevant into adulthood.

Collective qualitative findings highlight that body image in CF cannot be viewed solely through nutritional status, but must also be viewed as influenced by interpersonal support, healthcare communication, and shifting treatment realities and underline the need for empathetic, patient-centred communication in clinical encounters. Additionally, findings highlight the gap between provider recognition of body image concerns and their confidence in addressing them. Clinical practice should aim to normalise body image discussions within CF care and ensure that psychosocial specialists are available to support patients where needed. Future research should continue to explore the lived experiences of people living with CF, integrate the voices of people living with CF into intervention design, and evaluate approaches that combine functional, psychosocial, and appearance-related dimensions of body image.

### Strengths and limitations

To our knowledge, this is the first systematic review to synthesise both quantitative and qualitative evidence on body image among people living with CF in the era of CFTR modulators. A comprehensive search across multiple databases and preregistration in PROSPERO ensured methodological rigour and transparency. The use of the MMAT allowed quality appraisal across diverse study designs, while inclusion of qualitative findings added valuable contextual insight to the quantitative evidence. Nonetheless, some limitations should be acknowledged. Considerable heterogeneity in study design, measurement tools, and reporting precluded meta-analysis and limited comparability across studies. Most studies assessed body image indirectly *via* HRQoL instruments, which may underrepresent emerging concerns such as overweight and obesity in the modulator era. The limited number of qualitative and mixed-methods studies constrained the depth of experiential evidence available. Furthermore, gender-diverse people living with CF were not represented in any studies included in this review, indicating a need for future research to assess influences on body image among these individuals.

### Conclusion

This mixed-methods systematic review provides a comprehensive overview of body image in adults and young people living with CF in the context of evolving treatment paradigms. The findings indicate that body image disturbances remain prevalent and multifaceted, influenced not only by physical characteristics such as weight, BMI and body composition, but also by psychological wellbeing, social support, and functional health. While CFTR modulators have improved nutritional status and overall health outcomes, their impact on body image is complex. Evidence suggests that increases in weight and BMI may introduce new challenges to body image, particularly as existing assessment tools predominantly focus on undernutrition-related concerns.

Psychological factors, including anxiety and depression, emerged as consistent predictors of poorer body image, highlighting the importance of mental health in shaping self-perception. Similarly, social support and quality interpersonal relationships play a pivotal role in moderating body image experiences, underscoring the interdependence of psychosocial wellbeing and body satisfaction in people living with CF. Quantitative findings align with qualitative accounts, demonstrating that body image is closely tied to lived experiences, encompassing daily functioning, treatment burden, and social participation, rather than appearance alone. This reinforces the need for holistic, tailored strategies that integrate psychosocial support, functional rehabilitation, and patient-centred communication within clinical care.

Overall, this review highlights the evolving nature of body image in people living with CF and identifies a critical gap in current clinical practice, where body image concerns are often under-recognised and inadequately addressed. Future research should focus on developing and validating assessment tools suitable for the modulator era, as well as designing interventions that concurrently address appearance, function and psychosocial dimensions. Proactive identification and support for body image disturbances are essential to optimise the wellbeing, social participation, and overall quality of life of people living with CF in the new CF landscape.

**Points for clinical practice**

- Body image is a prevalent and evolving concern in the modulator era and is deeply intertwined with functional health, anxiety, depression, and social support.
- Findings reinforce the need for holistic, tailored strategies that integrate psychosocial support, functional rehabilitation, and patient-centred communication within clinical care.
- Current tools like the CFQ-R and CFQoL do not adequately capture overweight/obesity-related concerns and therefore are not appropriate to assess body image in the modulator era.
- Proactive identification and support for body image disturbances are essential to optimise the wellbeing, social participation, and overall quality of life of people living with CF in the new CF landscape.

## Supplementary material

**Please note:** supplementary material is not edited by the Editorial Office, and is uploaded as it has been supplied by the author. Appendix 1 ERR-0238-2025.SUPPLEMENT Appendix 2 ERR-0238-2025.SUPPLEMENT2 PRISMA 2020 checklist ERR-0238-2025.SUPPLEMENT3
