# Supplementary material for: Evolution of body image across treatment eras: a systematic review in young people and adults living with cystic fibrosis
Source: Eur Respir Rev. 2026 Apr 13;35(180):250238. doi: 10.1183/16000617.0238-2025 (PMC13169061; doi:10.1183/16000617.0238-2025)
Supplement: Supplementary file 1 [file ERR-0238-2025.SUPPLEMENT.pdf]

Appendix 1. Sample Search Strategy.

| Search | Query |
|---|---|
| 1. | "cystic fibrosis" |
| 2. | CF |
| 3. | Mucoviscidosis |
| 4. | "fibrocystic disease" |
| 5. | "Pancreas* Fibrosis" |
| 6. | "pancreas* cystic disease" |
| 7. | "Fibrocystic Disease of Pancreas" |
| 8. | Pancreas Fibrocystic Disease |
| 9. | "Cystic Fibrosis of Pancreas" |
| 10. | 1 or 2 or 3 or 4 or 5 or 6 or 7 or 8 or 9 ti, ab. |
| 11. | "physical appearance" |
| 12. | "thinness" |
| 13. | "fatness" |
| 14. | "body attitude*" |
| 15. | "shape concern" |
| 16. | "body concept" |
| 17. | "body precept*" |
| 18. | "body image*" |
| 19. | "body image/" |
| 20. | "body satisfaction" |
| 21. | "body ADJ2 dissatisfaction" |
| 22. | "body esteem" |
| 23. | "body ADJ2 concern" |
| 24. | "weight ADJ2 concern" |
| 25. | "appearance concern" |
| 26. | "weight preoccupation" |
| 27. | "weight perception" |
| 28. | muscularity |
| 29. | "body dysmorphi*" |
| 30. | "muscle dysmorphi*" |
| 31. | 11 or 12 or 13 or 14 or 15 or 16 or 17 or 18 or 19 or 20 or 21 or 22 or 23 or 24 or 25 or 26 or 27 or 28 or 29 or 30 ti,ab. |
| 32. | 10 and 31 |

*Sample search strategy for Medline (OVID) to examine the PICO (population, intervention, comparison, outcome) question: To evaluate and collate quantitative and qualitative reports of body image disturbances in adults and young people (≥16 years) with Cystic Fibrosis (CF) from observational and intervention studies.
